# Quantitative PCR Evaluation of Cellular Immune Responses in Kenyan Children Vaccinated with a Candidate Malaria Vaccine

**DOI:** 10.1371/journal.pone.0008434

**Published:** 2009-12-23

**Authors:** Jedidah Mwacharo, Susanna J. Dunachie, Oscar Kai, Adrian V. S. Hill, Philip Bejon, Helen A. Fletcher

**Affiliations:** 1 Centre for Geographical Medical Research, Kenya Medical Research Institute, Kilifi, Kenya; 2 The Jenner Institute, Nuffield Department of Clinical Medicine, University of Oxford, Churchill Hospital, Oxford, United Kingdom; World Health Organisation, Switzerland

## Abstract

**Background:**

The T-cell mediated immune response plays a central role in the control of malaria after natural infection or vaccination. There is increasing evidence that T-cell responses are heterogeneous and that both the quality of the immune response and the balance between pro-inflammatory and regulatory T-cells determines the outcome of an infection. As Malaria parasites have been shown to induce immunosuppressive responses to the parasite and non-related antigens this study examined T-cell mediated pro-inflammatory and regulatory immune responses induced by malaria vaccination in children in an endemic area to determine if these responses were associated with vaccine immunogenicity.

**Methods:**

Using real–time RT- PCR we profiled the expression of a panel of key markers of immunogenecity at different time points after vaccination with two viral vector vaccines expressing the malaria TRAP antigen (FP9-TRAP and MVA-TRAP) or following rabies vaccination as a control.

**Principal Findings:**

The vaccine induced modest levels of IFN-γ mRNA one week after vaccination. There was also an increase in FoxP3 mRNA expression in both TRAP stimulated and media stimulated cells in the FFM ME-TRAP vaccine group; however, this may have been driven by natural exposure to parasite rather than by vaccination.

**Conclusion:**

Quantitative PCR is a useful method for evaluating vaccine induced cell mediated immune responses in frozen PBMC from children in a malaria endemic country. Future studies should seek to use vaccine vectors that increase the magnitude and quality of the IFN-γ immune response in naturally exposed populations and should monitor the induction of a regulatory T cell response.

## Introduction

Global mortality and morbidity from *Plasmodium falciparum* malaria is high and has risen over the last 20 years, with an estimated minimum of one million deaths per year [1]. In sub Saharan Africa the greatest burden lies in children under the age of five [2], [3], [4]. Proper implementation of malaria control methods such as highly effective drugs and insecticide-treated nets could reduce mortality significantly [5]. However, it seems unlikely that these measures alone will halt the rising mortality of the disease. The development of an effective malaria vaccine is viewed as an effective long term strategy in reducing the burden of this disease [6]. Although natural immunity to *P. falciparum* malaria is short lived [7], strain-specific and develops after repeated exposure [8], immunisation experiments with irradiated sporozoites have consistently provided sterile immunity in experimental animals [9] and human volunteers [10], [11]. Immunity does not correlate to antibody titres against sporozoites and was not generated by sporozoites made incapable of invading hepatocytes by over-irradiation [11 12]. T-cell mediated immune responses play an important role in the control of malaria after natural infection or after vaccination. In mice, T-cell clones from irradiated sporozoite immunised mice transfer protective immunity to live sporozoite challenge [12]. In a large case-control study of Gambian children the HLA-B*53 allele was associated with protection against severe malaria [13], suggesting a role for CD8 positive T-cells.

Both pro-inflammatory and regulatory responses to malaria infection are important in determining the outcome of the disease. Chronic infection with malaria parasites has been associated with immunosuppressive responses to the parasite and to unrelated antigens [14].In animal models of malaria, the release of interferon gamma (IFN-γ) has been shown to mediate protection when secreted by *Plasmodium* specific CD8 [15] or CD4 T-cells [16], [17]. Following experimental malaria infection of human volunteers, enhanced Transforming Growth Factor beta (TGF-β1) activity was associated with suppression of pro-inflammatory cytokine responses, faster parasite growth, and induction of CD4+CD25+FOXP3+ regulatory T-cells (Tregs) [18]. It has been suggested that Tregs activated during malaria infection suppress immunity to malaria, by aiding parasite evasion of host immune responses [19] and may negatively affect naturally acquired immunity to malaria [20].

In a phase 2b malaria vaccine trial we evaluated the efficacy of a viral vector vaccine based prime-boost regime (FFM ME-TRAP), comprising of two vaccinations with an attenuated fowlpox virus (FP9) then one vaccination with modified vaccinia virus Ankara (MVA), both recombinant for a pre-erythrocytic antigen construct containing a multiple-epitope string and thombospondin-related adhesion protein (ME-TRAP). The trial recruited 1–6 year old children in the Kilifi district, Kenya and the end-point of the efficacy trial was clinical malaria. A rabies vaccine was used as the control in this trial. Over 18 months follow-up, there was no evidence that FFM ME-TRAP provided protection against malaria [21], [22]. In another study a DNA ME-TRAP and MVA ME-TRAP vaccine regimen was ineffective at reducing the natural infection rate in semi-immune African adults in the Gambia [23]. Although the reasons for this lack of efficacy are unclear, it has been shown that naturally acquired T-cell responses to Pf TRAP involve the release of competing pro- and anti-inflammatory cytokines [24].

This study aimed to look at T-cell mediated pro-inflammatory and regulatory immune responses induced by vaccination in children in a malaria endemic area to examine whether regulatory responses could be influencing vaccine immunogenicity in this setting.

## Results

### Gene Expression in FFM ME-TRAP Vaccinated Versus Rabies Vaccinated Children

A total of 88 PBMC samples from 30 children, obtained at different time points, were thawed for RNA extraction. In the FFM ME-TRAP group there were no significant differences in the characteristics of the included versus excluded study subjects (Table 1). As children with an immune response >100 SFC/10^6^ had been preferentially selected for analysis, the IFN-γ ELISPOT immunogenicity was significantly higher in selected versus excluded subjects. Due to the small size of the rabies vaccine group (n = 10) not all features of excluded subjects were represented by the included subjects (Table 1).

**Table 1:** Description of subjects selected for qPCR study.

Category	Covariate	Vaccine	Excluded	Included
**village**	Mapawa	Rabies	31 (16%)	1 (10%)
		FFM ME-TRAP	37 (20.4%)	2 (10%)
	Junju	Rabies	52 (26.8%)	2 (20%)
		FFM ME-TRAP	50 (27.6%)	5 (25%)
	Mwembe	Rabies	16 (8.2%)	0
		FFM ME-TRAP	14 (7.7%)	1 (5%)
	Gongoni	Rabies	43 (22%)	0
		FFM ME-TRAP	32 (26.5%)	7 (25%)
	kolewa	Rabies	52 (26.8%)	7 (70%)
		FFM ME-TRAP	48 (26.5%)	5 (25%)
**Age**	Mean Age	Rabies	3.6	3.1
	(years)	FFM ME-TRAP	3.6	3.7
**Weight**	Mean Weight	Rabies	12.8	11.6
	(kg)	FFM ME-TRAP	13.3	13.2
**Immunogenicity**	IFN-γ ELISPOT	Rabies	1.3	2.1
	(log SFC/10^6^)	FFM ME-TRAP	1.7	2.3
**Parasitemia**	week 1-month 3	Rabies	59 (30.4%)	2 (20%)
		FFM	68 (37.6%)	9 (45%)

**Figure 1:**
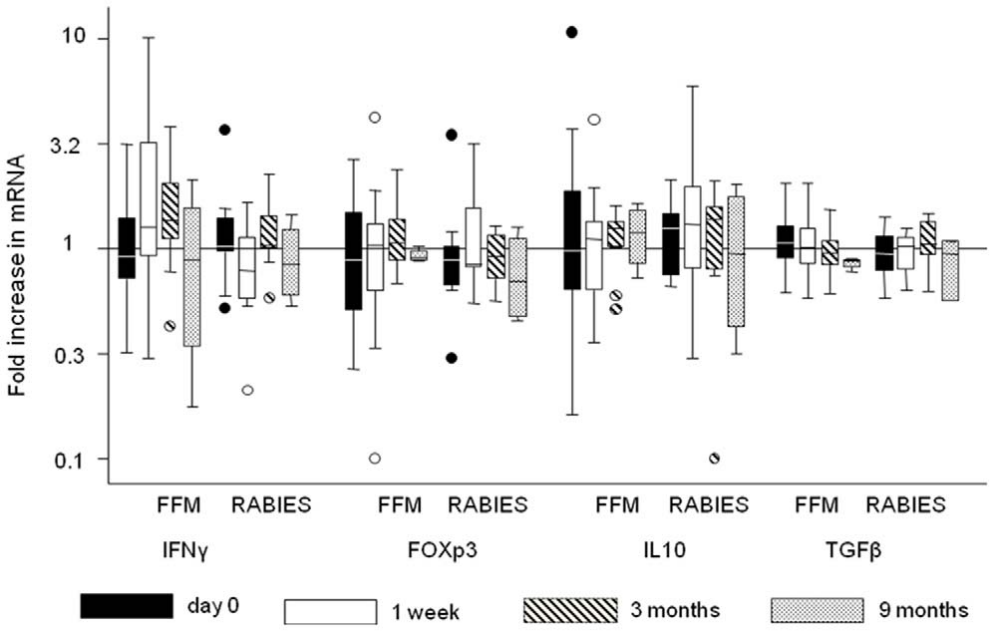
Gene expression analysis over time. Data are presented as a ratio of TRAP stimulated response over media stimulated response (mean+95%CI, n = 4–20). Vaccination by FFM ME-TRAP induced IFN-γ at 1 week (p = 0.0246) and at 3 months (p = 0.0913) after vaccination. No significant change in gene expression was observed at any time point post vaccination in the Rabies vaccine group.

We profiled vaccine responses by measuring mRNA of IFN-γ, FOXP3, IL-10 and TGF-β1 using real-time RT-PCR and we then compared gene expression between the FFM ME-TRAP and the Rabies vaccine groups (Figure 1). In the FFM ME-TRAP group IFN-γ levels showed a trend to elevation at 1 week and 3 months after the last dose of vaccine with the increased responses significant at 1 week (*P* = 0.02) when compared with baseline levels. At 9 months IFN-γ expression had returned to baseline levels. FOXP3 showed a trend to increase at 3 months but these responses were not significant (*P* = 0.2471) when compared with the baseline levels. IL-10 and TGF-β1 showed minor differences in expression across the different time points. In the Rabies group there were no significant changes in the expression of any gene tested at any time point. When the responses were compared between the two groups, only IFN-γ expression at 1 week showed significant response to the vaccine (Table 2).

**Table 2:** Mean expression of the different cytokines/markers at different time points (log copy number/HPRT).

		FFM-ME-TRAP		Rabies		
Cytokine	Time	N	Mean(95%CI)	N	Mean(95%CI)	*P* value
IFN-γ	Day 0	20	1.9(0.8–1.37)	10	1.12(0.76–1.65)	0.44
	1 week	20	1.62(1.08–2.42)	10	0.77(0.5–1.18)	0.02
	Month 3	10	1.39(0.89–2.16)	10	1.11(0.86–1.44)	0.34
	Month 9	4	0.74(0.14–3.99)	4	0.86(0.42–1.74)	0.8
FOXp3	Day 0	19	0.88(0.64–1.21)	10	0.9(0.58–1.39)	0.94
	1 week	20	0.76(0.4–1.44)	10	1.07(0.74–1.55)	0.45
	Month 3	10	1.15(0.88–1.5)	10	0.88(0.72–1.1)	0.1
	Month 9	4	0.92(0.82–1.04)	4	0.73(0.33–1.62)	0.38
IL-10	Day 0	20	1.9(0.70–1.78)	10	1.12(0.84–1.48)	0.93
	1 week	20	0.95(0.71–1.27)	10	1.32(0.73–2.39)	0.23
	Month 3	10	1.08(0.83–1.41)	10	0.96(0.44–2.1)	0.74
	Month 9	4	1.137(0.63–2.04)	4	0.871(0.22–3.44)	0.59
TGF-β1	Day 0	20	1.057(0.91–1.2)	10	0.943(0.76–1.17)	0.34
	1 week	20	1.045(0.9–1.2)	10	0.939(0.8–1.1)	0.33
	Month 3	10	1.387(0.89–2.16)	10	1.112(0.86–1.44)	0.37
	Month 9	4	0.851(0.77–0.94)	3	0.836(0.36–1.95)	0.92

The mean mRNA expression levels were compared between the two vaccination groups.

**Figure 2:**
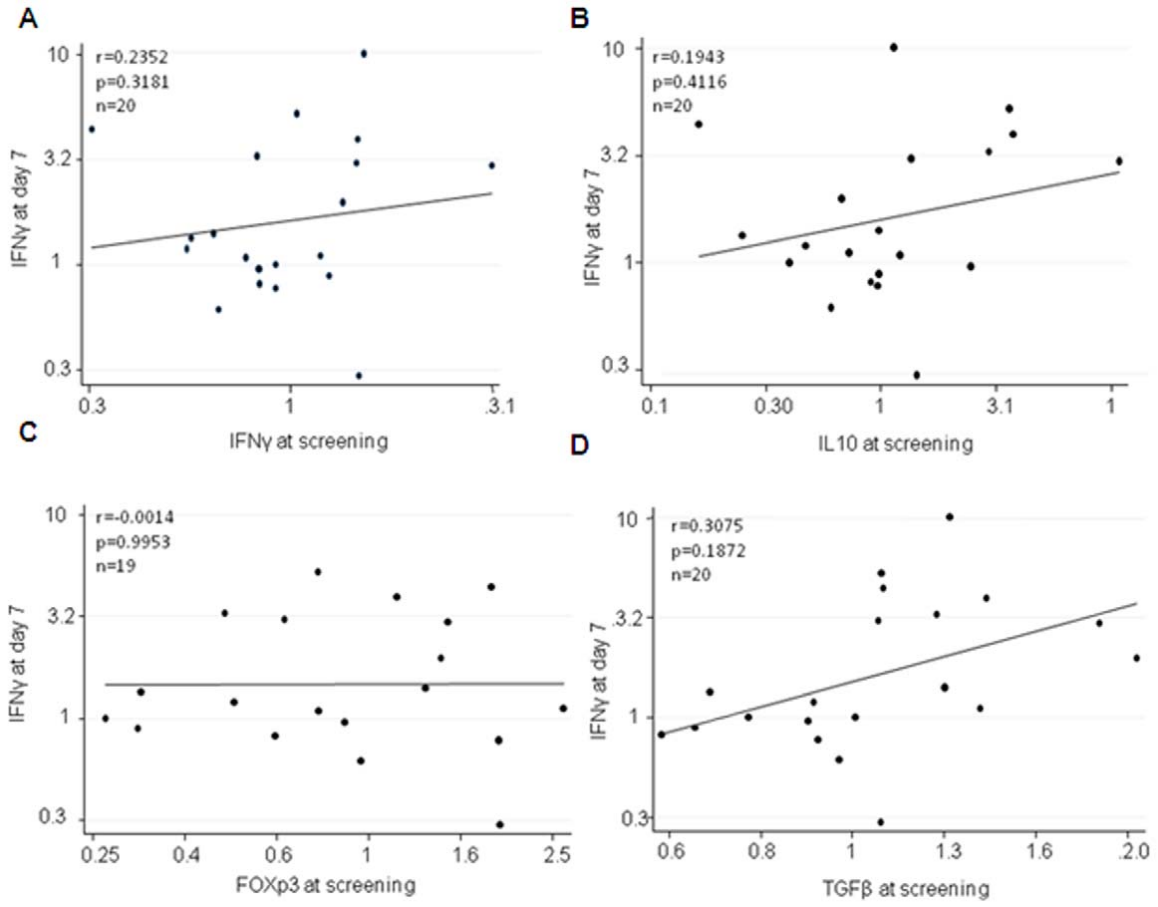
Correlation between IFN-γ mRNA 1 week following vaccination and baseline IL10, FoxP3 and TGF-β1 mRNA. There was no correlation between antigen specific IFN-γ and A) IFN-γ at screening B) IL10 at screening C) FoxP3 at screening D) TGFB at screening (n = 20).

FOXP3, TGF-β1 and IL-10 responses observed in the FFM ME- TRAP group were comparable to the ones observed in the Rabies vaccine group. There was no correlation between the baseline response of IL-10, FoxP3 or TGF-β1 and IFN-γ responses at 1 week following vaccination (Figure 2A-D).

### qPCR Detection of T Cell Clones

**Figure 3:**
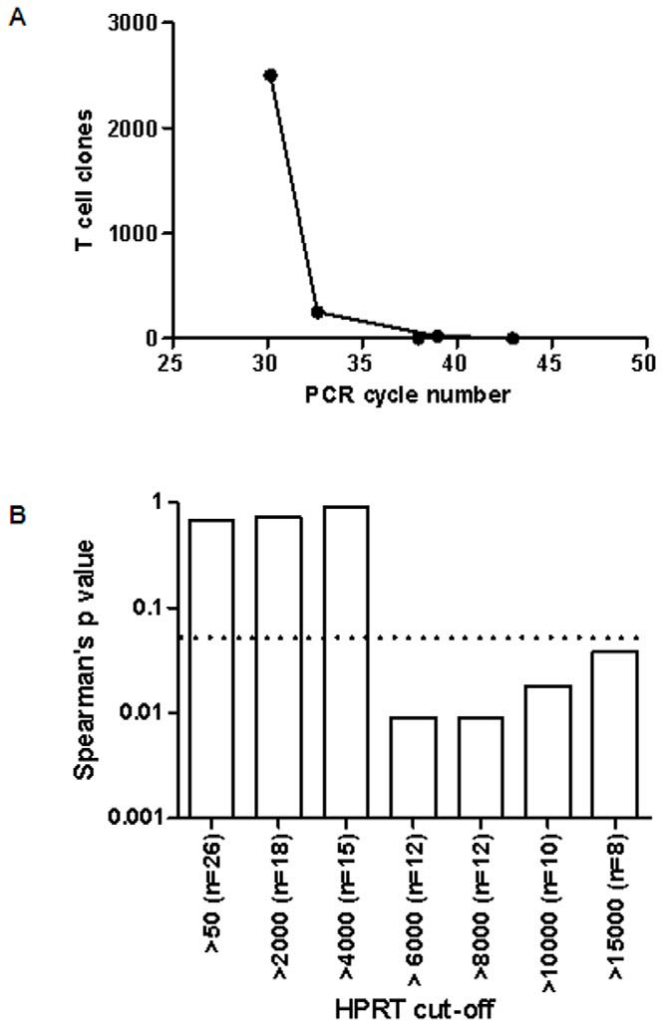
Correlation of qPCR with the IFN-γ ELISPOT assay is dependent on the frequency of antigen specific T cells present and the quantity of viable cells tested. A) The qPCR detection of IFN-γ secreted by a known number's of mouse CD8 T cell clone's. The detection limit is >25 and <250 T cell clones/10^6^ splenocytes. B) Spearman's correlation of TRAP peptide stimulated IFN-γ measured by qPCR and ELISPOT improves as the cut-off for HPRT copy number increases. The dashed line indicates a p value of 0.05.

Using a mouse T cell clone specific for the H-2Kd-restricted pb9 CTL epitope (SYIPSAEKI) from the circumsporozoite protein of *P. Berghei* we have found that we can detect 250 but not 25 T cell clones secreting IFN-γ in response to pb9 peptide stimulation when using the qPCR assay (Figure 3A). Using human PBMC we can detect 20 IFN-γ T cells/10^6^ PBMC using the human IFN-γ ELISPOT assay [25]. The qPCR assay may therefore be up to 10 times less sensitive for the detection of IFN-γ T secreting cells when compared to the ELISPOT assay.

### Correlation of qPCR and ELISPOT for the Detection of IFN-γ Is Influenced by HPRT Copy Number of the Tested Sample

To assess the quality of our qPCR data we sought to correlate IFN-γ mRNA expression with IFN-γ protein expression in these children as determined by the ELISPOT assay [26]. Although we had preferentially selected children with a positive ELISPOT response 4 of the 30 children selected had a response <100 SFC/10^6^ PBMC. These 4 children were excluded from the correlation analysis as we had determined that the sensitivity of our qPCR assay was between 25 and 250 SFC/10^6^ PBMC. Using the week 1 TRAP stimulated samples we then correlated IFN-γ mRNA and protein expression in the remaining 26 children. No significant correlation was observed using data from the 26 children (Figure 3B). HPRT is a house keeping gene expressed at a similar level in all cells. The copy number of HPRT is therefore an indication of the number of viable cells present in a sample and the range of HPRT values in our 26 children was from 54 to 80143 copies per sample. To determine if the inclusion of samples with low HPRT copy number could be adversely effecting the correlation between IFN-γ mRNA and protein we introduced a cut-off of HPRT copy number ranging from 50 to 15000 copies per sample. We found that using samples with an HPRT copy number >6000 we obtained a significant correlation between IFN-γ protein and mRNA expression (Figure 3B). These results indicate that a minimum HPRT copy number of 6000 and a minimum ELISPOT response of 100 SFC/10^6^ PBMC is required to demonstrate correlation of qPCR with ELISPOT responses.

### FoxP3 mRNA Expression Is Increased in Subjects Vaccinated with FFM ME-TRAP

**Figure 4:**
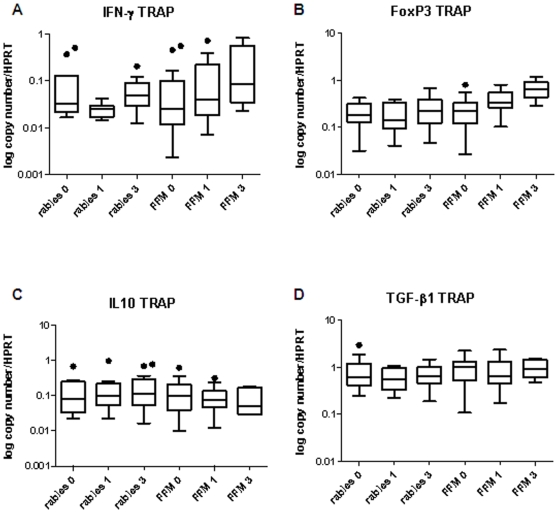
TRAP peptide specific IFN-γ and FoxP3 mRNA expression increase in the FFM ME-TRAP vaccine group over time. A) IFN-γ mRNA expression B) FoxP3 mRNA expression C) IL10 mRNA expression D) TGF-β1 mRNA expression (n = 10–20).

**Figure 5:**
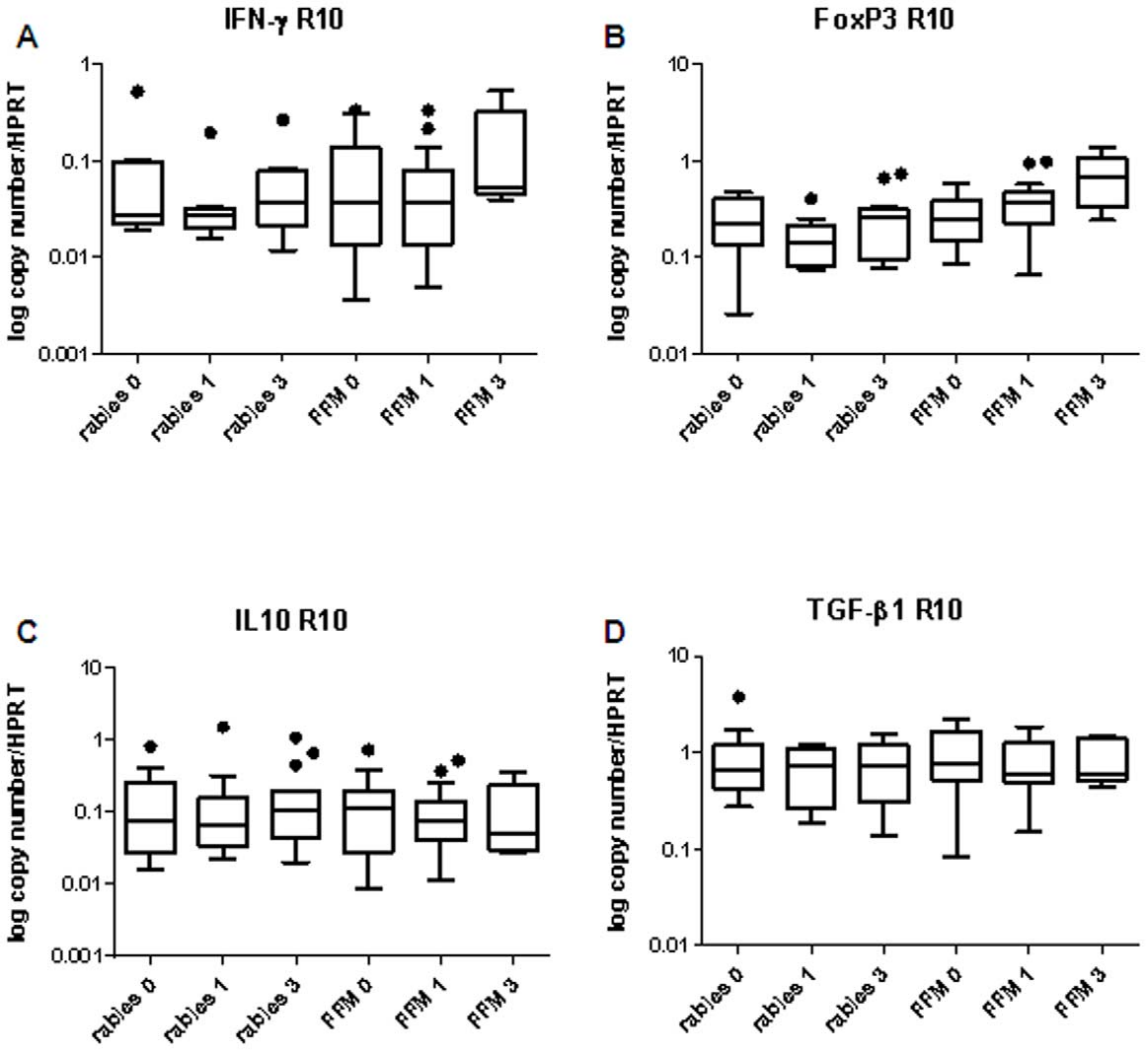
FoxP3 mRNA expression increases over the 3 month study period in media-stimulated cells from subjects in the FFM ME-TRAP vaccine group. A) IFN-γ mRNA expression B) FoxP3 mRNA expression C) IL10 mRNA expression D) TGF-β1 mRNA expression (n = 10–20).

The fold increase in TRAP peptide stimulated over media-stimulated gene expression showed only an increase in IFN-γ gene expression in the FFM ME-TRAP vaccinated group (Figure 1). We then performed a paired comparison of gene expression over time in both TRAP stimulated and media stimulated cells over the 3 month study period. There was a significant increase in IFN-γ gene expression in TRAP stimulated cells in the FFM ME-TRAP vaccinated group at week 1 when compared to screening (p = 0.016, Figure 4A). There was also a significant increase in FoxP3 mRNA expression at week 1 compared to screening in the FFM ME-TRAP vaccinated group (p = .0084, Figure 4B). FoxP3 expression at week 1 was significantly higher in the FFM ME-TRAP group when compared to the rabies group at week 1 (p = 0.016). We then examined gene expression over time in media stimulated cells. There was a significant increase in FoxP3 mRNA expression at week 1 compared to screening in the FFM ME-TRAP vaccinated group (p = .0149, Figure 5B). FoxP3 expression at week 1 was significantly higher in the FFM ME-TRAP group when compared to the rabies group at week 1 (p = .0068). When examining the fold change of FoxP3 expression in TRAP stimulated over media stimulated cells there appeared to be no change in gene expression following vaccination (Figure 1). However, a paired analysis of data over time reveals a non-antigen specific increase in FoxP3 expression over the study period (Figures 4B and 5B).

## Discussion

Understanding vaccine induced immune responses in malaria exposed populations is vital as previous malaria exposure may affect the quality and dynamics of vaccine immunogenicity.

This study demonstrates a method of analysing cellular responses to vaccinations that can be used on cryopreserved samples from field trials. Responses were studied following both experimental vaccination against malaria and a control vaccine.

We demonstrate real-time RT-PCR to be a sensitive way of detecting cellular responses in this setting. A modest but significant increase in IFN-γ expression was observed one week after last vaccination in the antigen-stimulated cells in subjects receiving the FFM regimen, and this is consistent with the responses observed by *ex vivo* ELISpot assays as reported earlier [21]. Although we were able to detect vaccine induced IFN-γ expression we have found that the IFN-γ ELISPOT assay is more sensitive than qPCR for the detection of IFN-γ secreting antigen specific T cells. The ELISPOT assay can reliably detect >20 SFC/10^6^ PBMC [25] whereas we could not detect 25 IFN-γ secreting T cells using the qPCR assay. We have also found that detection of an IFN-γ secreting T cell is dependent of the quantity of viable cells tested as measured using HPRT copy number. Although limiting the analysis to samples with an HPRT copy number >6000 did not alter the findings of our study (data not shown) we would use this cut-off as a quality control step in future qPCR studies. Our method is not as sensitive as ELISPOT for the detection of IFN-γ yet it enables the simultaneous detection of multiple genes and from a small number of cells and is therefore a useful tool for exploratory immunology in a field setting.

There are several known mechanisms for malaria induced immunosupression such as, impairment of dendritic cell function by malaria [27] and induction of T-cell regulatory responses by placental malaria at birth [28]. This could indicate causal association between reduced immunogenicity and more frequent malaria and other chronic infections. Although we did not see any direct correlation of anti inflammatory cytokines with vaccine immunogenicity we did observe an increase in FoxP3 expression in both TRAP stimulated and media stimulated cells in the FFM ME-TRAP vaccinated group from screening to one week following final vaccination. We did not observe an increase in FoxP3 expression in the rabies vaccinated group, however, there were fewer incidences of parasitaemia in the 10 rabies vaccinated children selected for our study than in the FFM ME-TRAP group. It is therefore possible that the increase in FoxP3 expression is driven by natural exposure to parasites rather than by FFM ME-TRAP vaccination. In an earlier study in this cohort, parasitaemia was associated with suppression of natural or vaccine-induced acquisition of IFN-γ T-cell responses [29]. Others have shown that elevated levels of FoxP3 expressing regulatory T cells are found in malaria infected patients [30]. Although it is likely that increased FoxP3 expression in our study is linked to natural exposure to parasite further studies are warranted to exclude the possibility of the effect being exacerbated by malaria vaccination.

Over nine months follow-up during the original study there was a non-significant increase in the incidence of malaria in children who received FFM ME-TRAP [26], although this was not sustained during an extended 18 month period of follow-up [22]. The current study found no evidence for enhanced production of the anti-inflammatory cytokines TGF-β1 and IL-10 in the FFM ME-TRAP group when compared to rabies vaccinated controls. There is increasing evidence that vaccine efficacy is determined by not only the magnitude but the quality of the vaccine induced T cellular immune response [31], [32]. The magnitude of the IFN-γ response induced by vaccination in our study was small. It is possible that immune regulatory mechanisms are limiting the T cellular immune response in naturally exposed individuals. Further investigation of regulatory T cell responses in both malaria vaccinated and non-vaccinated children are warranted,

### Conclusions

Measurement of gene expression of a panel of key cytokines over several timepoints by real-time RT-PCR provides a method of monitoring cellular responses to vaccination, and represents a method for detailed analysis of samples obtained in a field setting.

We have seen some evidence for the induction of a FoxP3 expression in children vaccinated with FFM ME-TRAP. To obtain a malaria vaccine that will be useful in Africa, further vaccine studies should seek to increase the magnitude and quality of the IFN-γ immune response and investigate the induction of regulatory T cell responses in naturally exposed populations.

## Materials and Methods

### Ethics Statement

This study was performed with the permission of KEMRI National Ethics Committees, and COREC, the NHS Central Office for Research Ethics Committees. The trial was assigned registration number ISRCTN88335123 with the International Standard Randomized Controlled Trial Number Register (http://www.controlled-trials.com/isrctn/trial/. After a series of public meetings and individual discussions parents or guardians of the participants were assigned a screening date. On the day of screening study information was repeated to parents or guardians and written informed consent was obtained before study procedures were initiated.

### Study Participants

Frozen samples were obtained from children recruited in a phase 2b malaria vaccine trial that evaluated the efficacy of the regimen of FFM ME-TRAP in preventing episodes of clinical malaria among 1–6 year old children in Kilifi, Kenya. Rabies vaccine was used as a control. Peripheral blood mononuclear cells (PBMCs) were isolated and frozen as previously described [21] at screening, 1 week, 3 and 9 months after last vaccination. Children for inclusion in the study were selected on the basis of the IFN-γ ELISPOT response (response range 2.5–512.5 SFC/10^6^ PBMC). Children with an IFN-γ ELISPOT response >100 SFC/10^6^ PBMC were preferentially selected for inclusion in the study as these children were most likely to have a detectable change in gene expression as measured by qPCR.

### PBMC Stimulation

PBMCs were thawed and re-suspended at 10 million cells per ml of media (RPMI with 10% fetal calf serum [Sigma-Aldrich, Poole, Dorset, UK] and Penicillin-streptomycin (Invitrogen, Paisley, UK). 1.0×10^6^ cells in 100 µl of media were transferred in duplicate wells to a 96-well plate. Cells were incubated in a 37°C/5% CO_2_ incubator for 4 hours, then stimulated by adding 100 µl of TRAP peptide pools (TRAP derived 20-mer peptides overlapping by 10 amino acids [33]) at 4 µg/ml (final concentration 2 µg/ml) or in 100 µl of media alone. The cultures were incubated for a further 12 hours. Stimulation was stopped by spinning at 1200 rpm for 5 minutes. The supernatant was discarded and 100 µl of RNeasy RTL buffer (Qiagen, Crawley, UK) with 10 µl beta-mercaptoethanol (VWR, Lutterworth, Leicestershire, UK)/ml added. Cells were then stored at −20°C before RNA extraction.

### RNA Extraction

RNA was extracted from 1×10^6^ PBMC using Rneasy® Mini kit (Qiagen, Crawley, UK) according to the manufacturer's instructions.

Reverse Transcription

Reverse transcription of mRNA was performed using oligo-dt (MWG-Biotech Milton Keynes,UK) and the OmniscriptRT® Kit (Qiagen, Crawley, UK). Briefly, 5 µl of RNA extracted was added to 15 µl of the reaction mix in a 1.5 ml eppendorf tube. This was then incubated for 1–2 hours at 37°C followed by 5 mins at 94°C.

Real-Time PCR

1 µl of the template cDNA was added into the master mix with the relevant primer make a final volume of 20 µl. Each of the samples was run in duplicate with 2 negative controls included in each PCR run. Hypoxanthine phosphoribosyl transferase (HPRT) was used as the house-keeping gene and PCR data was normalised against HPRT. Real -Time PCR was performed using LightCycler® (Roche) with QuantiTect®SYGR® Green kit (Qiagen, Crawley, UK). Primers were targeted towards the following sequences;

FOXP3 (F 5′-CACTTACAGGCACTCCTCCAGG-3′ and R 5′-CACCGTTGAGAGCTGGTGCAT-3′), TGF-β1 (F5′-GGACATCAACGGGTTCAC T-3′ and R 5′-CCGGTTCATGCCATGAATGG-3′), IFN-γ (F 5′-ATTCGGTAACTGACTTGAATGTCC-3′ and R 5′-CTCTTCGACCTCGAAACAGC-3′), IL-10 (F 5′-GGCCGTGGAGCAGGT-3′ and R 5′-CACTCATGGCTTTGTAGATGCC-3′), HPRT (F 5′-TATGGACAGGACTGAACGTC-3′ and R 5′-CTACAATGTGATGGCCTCCC-3′) mouse IFN-γ(F 5′-GGGTTGTTGACCTCA AACTTGGCA-3′ and R 5′-CAGGCCATCAGCAACAACAT-3′).

Cycling conditions of an initial activation step of 15 min at 95° followed by 45 cycles of 30 s at 94°, 30 s at 60° and 1 min at 72°were used for each primer pair.

### qPCR Detection of T Cell Clones

To determine the minimum number of IFN-γ secreting T cells that could be detected by the qPCR assay a mouse T cell clone specific for the H-2Kd-restricted pb9 CTL epitope (SYIPSAEKI) from the circumsporozoite protein of *P. berghei* was expanded using pb9 peptide loaded irradiated splenocytes from naive BALB/c mice. After 7 days culture in MEM medium (Sigma Aldrich) supplemented with 10% heat-inactivated FCS, 4 mM L-glutamine, 100 U/ml penicillin, 100 mg/ml streptomycin sulfate, 100 µM β-mercaptoethanol and 10 U/ml IL-2 (Lymphocult HT, Biotest) expanded T cell clones were washed and counted. A 10 fold dilution series of pb9 T cell clones was prepared to obtain 250,000 to 2.5 T cell clones. T cell clones were mixed with naive splenocytes to obtain a total cell number of 1×10^6^. Cells were then stimulated overnight with 10 µg/ml pb9 peptide, pelleted and then RNA extracted for qPCR analysis as described above.

### Statistical Analysis

The mean copy number for each of genes was normalised against the HPRT copy number for both TRAP stimulated and media stimulated cells. The TRAP stimulated value was then divided by media stimulated value to give a fold change ratio. Geometric means (95% CI) were calculated from fold change ratio at each time point. Statistical analysis was performed on log transformed values using Student's *t* test. *P*<0.05 was considered significant. The results in the graphs are expressed as log values (Stata 9™, Stata coorp). Paired analysis of data points over time were performed using Wilcoxon signed rank and correlations were performed using Spearman's rho (SPSS Statistics 17.0).
