# Correction: Quantitative PCR Evaluation of Cellular Immune Responses in Kenyan Children Vaccinated with a Candidate Malaria Vaccine

**DOI:** 10.1371/annotation/9a1d1873-2f7e-4d69-a1b0-8e3797e8093a

**Published:** 2011-02-11

**Authors:** Jedidah Mwacharo, Susanna J. Dunachie, Oscar Kai, Adrian V. S. Hill, Philip Bejon, Helen A. Fletcher

There is an error in the financial disclosure. The correct funding for Philip Bejon is: "P. Bejon is supported by the NIHR Biomedical Research Centre Oxford."

